# Safety and efficacy of human polymerized hemoglobin on guinea pig resuscitation from hemorrhagic shock

**DOI:** 10.1038/s41598-022-23926-y

**Published:** 2022-11-28

**Authors:** Cynthia R. Muller, Alexander T. Williams, Cynthia Walser, Allyn M. Eaker, Jose Luis Sandoval, Clayton T. Cuddington, Savannah R. Wolfe, Andre F. Palmer, Pedro Cabrales

**Affiliations:** 1 Department of Bioengineering, University of California, 0412, 9500 Gilman Dr. La Jolla, San Diego, CA 92093-0412 USA; 2 William G. Lowrie Department of Chemical and Biomolecular Engineering, The Ohio State University, Columbus, OH USA

**Keywords:** Trauma, Physiology

## Abstract

For the past thirty years, hemoglobin-based oxygen carriers (HBOCs) have been under development as a red blood cell substitute. Side-effects such as vasoconstriction, oxidative injury, and cardiac toxicity have prevented clinical approval of HBOCs. Recently, high molecular weight (MW) polymerized human hemoglobin (PolyhHb) has shown positive results in rats. Studies have demonstrated that high MW PolyhHb increased O_2_ delivery, with minimal effects on blood pressure, without vasoconstriction, and devoid of toxicity. In this study, we used guinea pigs to evaluate the efficacy and safety of high MW PolyhHb, since like humans guinea pigs cannot produce endogenous ascorbic acid, which limits the capacity of both species to deal with oxidative stress. Hence, this study evaluated the efficacy and safety of resuscitation from severe hemorrhagic shock with high MW PolyhHb, fresh blood, and blood stored for 2 weeks. Animals were randomly assigned to each experimental group, and hemorrhage was induced by the withdrawal of 40% of the blood volume (BV, estimated as 7.5% of body weight) from the carotid artery catheter. Hypovolemic shock was maintained for 50 min. Resuscitation was implemented by infusing 25% of the animal’s BV with the different treatments. Hemodynamics, blood gases, total hemoglobin, and lactate were not different before hemorrhage and during shock between groups. The hematocrit was lower for the PolyhHb group compared to the fresh and stored blood groups after resuscitation. Resuscitation with stored blood had lower blood pressure compared to fresh blood at 2 h. There was no difference in mean arterial pressure between groups at 24 h. Resuscitation with PolyhHb was not different from fresh blood for most parameters. Resuscitation with PolyhHb did not show any remarkable change in liver injury, inflammation, or cardiac damage. Resuscitation with stored blood showed changes in liver function and inflammation, but no kidney injury or systemic inflammation. Resuscitation with stored blood after 24 h displayed sympathetic hyper-activation and signs of cardiac injury. These results suggest that PolyhHb is an effective resuscitation alternative to blood. The decreased toxicities in terms of cardiac injury markers, vital organ function, and inflammation following PolyhHb resuscitation in guinea pigs indicate a favorable safety profile. These results are promising and support future studies with this new generation of PolyhHb as alternative to blood when blood is unavailable.

## Introduction

Trauma remains one of the leading causes of death worldwide, with approximately half of all trauma deaths attributed to hemorrhage^1^. After hemorrhagic shock (HS), blood pressure (BP) drastically decreases due to lost blood volume, limiting blood perfusion and oxygen (O_2_) delivery to tissues, and fails to satisfy cellular and tissue metabolic O_2_ needs^2^. Currently, whole blood and blood components are the gold standard for resuscitation of patients after HS, but blood has a short shelf life and limited availability, and the use of ex vivo stored blood and stored red blood cells (RBCs) can be detrimental and increase mortality rates in certain scenarios^3,4^. Blood shortages are common, and the frequency of these shortages is expected to increase in the future based on current donation rates and blood usage trends^5^. While elective surgeries can be delayed during blood shortages, in emergencies such as trauma, the need for blood is immediate. Furthermore, blood availability can be impacted during natural disasters, wars, and more recently during the COVID-19 pandemic, highlighting the importance of developing alternatives to blood transfusion.

Numerous studies have demonstrated that the quality of whole blood and RBCs decreases as ex vivo storage time increases^6,7^. The maximum time approved by the United States Food and Drug Administration (FDA) for ex vivo storage of RBCs is 42 days and whole blood is 21 days at 4 °C in the appropriate additive solution^8^. In an attempt to decrease RBC, whole blood or blood component transfusion dependence, crystalloids have been extensively studied for the management of HS, but treatment with these solutions has limitations such as hemodilution, blood acidification, coagulopathy, and most importantly they dilute and reduce the O_2_ capacity of blood^9^. New strategies for the treatment of HS suggest appropriate treatment should focus on restoring tissue O_2_ delivery by restoring blood volume (BV) and blood flow in both the macro and microcirculation^10,11^. Therefore, ideal HS resuscitation fluids should have certain characteristics such as significant O_2_ carrying capacity, preservation of hemostasis, universal compatibility, and preservation of blood biophysical properties such as osmolarity, colloidal oncotic pressure, and blood viscosity^2^.

Hemoglobin (Hb) based O_2_ carriers (HBOCs) have been developed with promising results as alternatives to blood, when blood is not available. However, previous generations of HBOCs have failed in clinical trials, as they lead to vasoconstriction, systemic hypertension, and cardiac lesions^12^. Recently, we have developed and evaluated a high molecular weight (MW) polymerized human Hb (PolyhHb) as an alternative to blood. Increasing the MW of the PolyhHb appears to resolve the issues associated with vasoactivity, thus mitigating vasoconstriction and hypertension previously observed in earlier generations of HBOCs^13,14^. High MW PolyhHb has also been shown to be safer and well-tolerated at higher doses, and capable of preserving microvascular blood flow and increasing O_2_ delivery with limited toxicity^13,14^. Moreover, our most recent publication demonstrated that a high MW PolyhHb can restore hemodynamics to a similar degree as blood in a severe model of traumatic brain injury followed by HS in rats^15^.

However, preclinical toxicological evaluation of HBOCs requires the use of an appropriate animal species that closely reflects human physiology and pharmacology. Interestingly, evaluation of early generation HBOCs in rats and mice failed to detect toxicity and cardiac ischemia later observed in clinical trials^16,17^. Recently, this issue was attributed to the fact that rats and mice produce endogenous ascorbic acid (AA), and thus possess a different antioxidant status compared to humans, since humans cannot produce AA and must obtain it from their diet^16,17^. AA is the primary small-molecule reducing agent in blood that maintains the non-oxidized ferrous (HbFe^2+^) form of Hb/HBOCs, and prevents accumulation of the non-O_2_ carrying and heme unstable methemoglobin (metHb) (HbFe^3+^) form of Hb/HBOCs^16,17^. Consequently, guinea pigs may be a more useful small animal species to estimate the safety of new HBOCs, since guinea pigs, like humans, are incapable of endogenous production of AA due to the evolutionary loss of functional hepatic L-gulonolactone oxidase (LGO)^17^. Therefore, this study aimed to evaluate the efficacy and safety of resuscitation from HS with high MW PolyhHb compared to fresh blood and 2-week-old stored blood, in a guinea pig model. Our hypothesis was that high MW PolyhHb is safe and effective at restoring hemodynamics after resuscitation from HS in guinea pigs.

## Results

### Hemodynamics and hematological parameters

**Figure 1:**
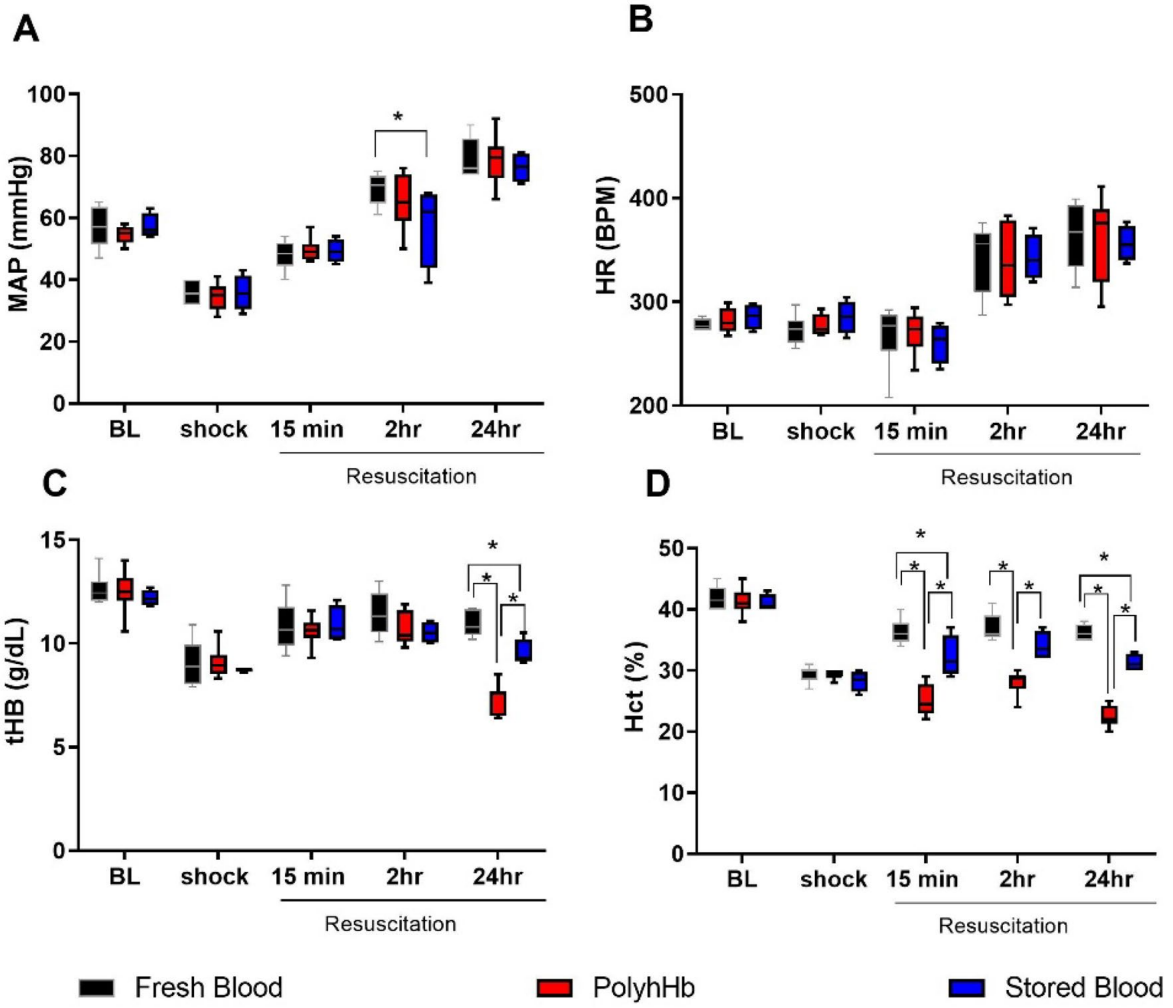
Hemodynamics and hematological parameters. PolyhHb restored hemodynamics after hemorrhagic shock (HS) similar to fresh and stored blood. However, stored blood took a longer period of time to achieve a similar blood pressure compared to fresh blood and PolyhHb. Although, PolyhHb half-life was approximately 13 h and total hemoglobin was lower 24 h after resuscitation, PolyhHb restored hemodynamics at 24 h post-resuscitation. (**a**) Mean arterial pressure (MAP); (**b**) Heart rate (HR); (**c**) Total hemoglobin (tHb); (**d**) Hematocrit (Hct). Measurements were taken at baseline (BL), 50 min into hemorrhagic shock (shock) and 15 min, 2 h, and 24 h after resuscitation. Animals were awake at the 2 h and 24 h timepoints. *, *p* < 0.05. Statistical comparisons were completed using a two way- ANOVA; Tukey’s multiple comparison test.

Mean arterial pressure (MAP), heart rate (HR), total Hb (tHb) and hematocrit (Hct) are presented in Fig. 1. All groups experienced a similar decrease in MAP during HS (approximately 35% compared to baseline), and there was no difference in MAP between groups at 24 h after resuscitation. However, two hours after resuscitation, the stored blood (SB) group had lower MAP (57.8 ± 6.6 mmHg) compared to the fresh blood group (FB) (*p* = 0.01), while the MAP for the PolyhHb group (64.6 ± 3.4 mmHg) was no different from the FB group (69.3 ± 2.1 mmHg). After 24 h, all groups restored MAP to a similar level (FB: 79.0 ± 3.0; PolyhHb: 79.0 ± 2.8; SB: 76.3 ± 2.5 mmHg). There were no differences in HR between groups at any of the timepoints. The differences in HR between the procedure day (hemorrhagic shock resuscitation) and the 24-h time point are due to the effects of anesthesia on the animals during the procedures, and the animals being awake at the 24-h time point.

The tHb decreased in all groups similarly after hemorrhage, and was restored to a similar degree in all groups at 15 min and 2 h after resuscitation. However, the PolyhHb group had statistically lower tHb (7.4 ± 0.3 g/dL) compared to the FB group (11.0 ± 0.3 g/dL, *p* < 0.01) and to the SB group (9.6 ± 0.3 g/dL, *p* < 0.01) 24 h after resuscitation. The Hct decreased in all groups similarly after hemorrhage, by approximately 12% from baseline across all groups during HS. Resuscitation with FB restored the Hct (36 ± 0.8%) significantly higher than resuscitation with SB (32 ± 1.7%) at 15 min (*p* = 0.01). The Hct was not recovered in the group resuscitated with PolyhHb (25 ± 0.9%), and was statistically lower compared to FB (*p* < 0.01) and SB (*p* < 0.01).

Blood gases and lactate are presented in Table 1. There were no statistically significant differences between groups in terms of pH, pO_2_, or pCO_2_ throughout the experimental protocol. Classically, it is expected to observe increased lactate concentrations after HS^18^, since hypoxic tissues change energy production to anaerobic metabolism, resulting in higher lactate levels. In the present study, the lactate concentration increased equally between groups during HS and recovered to baseline for all groups at 24 h after resuscitation (Table 1).

**Table 1:** Blood gases, pH and lactate levels.

		Fresh blood (FB)	PolyhHb	Stored blood (SB)
pCO_2_ (mmHg)	BL	48.8 ± 2.9	50.5 ± 1.8	53.3 ± 5.7
	Shock 50 min	41.9 ± 1.8	43.5 ± 1.5	45.6 ± 3.9
	Resus 15 min	44.2 ± 2.3	40.5 ± 2.1	49.9 ± 3.2
	Resus 2 h	38.8 ± 1.9	29.6 ± 1.3	36.3 ± 3.0
	Resus 24 h	36.0 ± 2.8	39.0 ± 1.0	37.1 ± 1.9
pO_2_ (mmHg)	BL	107.4 ± 3.8	88.6 ± 10.6	108.6 ± 5.8
	Shock 50 min	106.2 ± 2.8	92.1 ± 8.6	103.9 ± 5.5
	Resus 15 min	95.1 ± 5.8	88.3 ± 9.5	98.7 ± 4.1
	Resus 2 h	82.0 ± 6.7	84.1 ± 7.9	82.2 ± 6.4
	Resus 24 h	75.2 ± 8.5	72.3 ± 3.6	67.3 ± 6.8
pH	BL	7.262 ± 0.033	7.290 ± 0.016	7.199 ± 0.068
	Shock 50 min	7.316 ± 0.044	7.313 ± 0.017	7.355 ± 0.043
	Resus 15 min	7.343 ± 0.032	7.341 ± 0.021	7.341 ± 0.037
	Resus 2 h	7.435 ± 0.018	7.429 ± 0.019	7.445 ± 0.034
	Resus 24 h	7.436 ± 0.017	7.456 ± 0.017	7.449 ± 0.015
Lactate (mmol/L)	BL	1.94 ± 0.75	1.47 ± 0.39	1.63 ± 0.39
	Shock 50 min	3.56 ± 1.18	2.66 ± 0.47	2.07 ± 0.38
	Resus 15 min	1.86 ± 0.78	1.90 ± 0.39	1.23 ± 0.19
	Resus 2 h	0.76 ± 0.04	2.52 ± 0.57	0.87 ± 0.17
	Resus 24 h	1.08 ± 0.48	1.19 ± 0.16	0.67 ± 0.03

All resuscitation solutions preserved blood gases and pH within physiological ranges after resuscitation from HS. Moreover, all resuscitation solutions restored lactate to normal levels 24 h after resuscitation. Animals were awake at the 2 h and 24 h timepoints. Data was presented as the mean ± SE. BL (Baseline); HS (Hemorrhagic Shock); Resus (Resuscitation). No statistical differences were detected between groups in blood gases, blood pH, or blood lactate. Statistical comparisons were completed using a two way-ANOVA; Tukey’s multiple comparison test.

### Vital organ damage and injury

#### Liver and kidney

**Figure 2:**
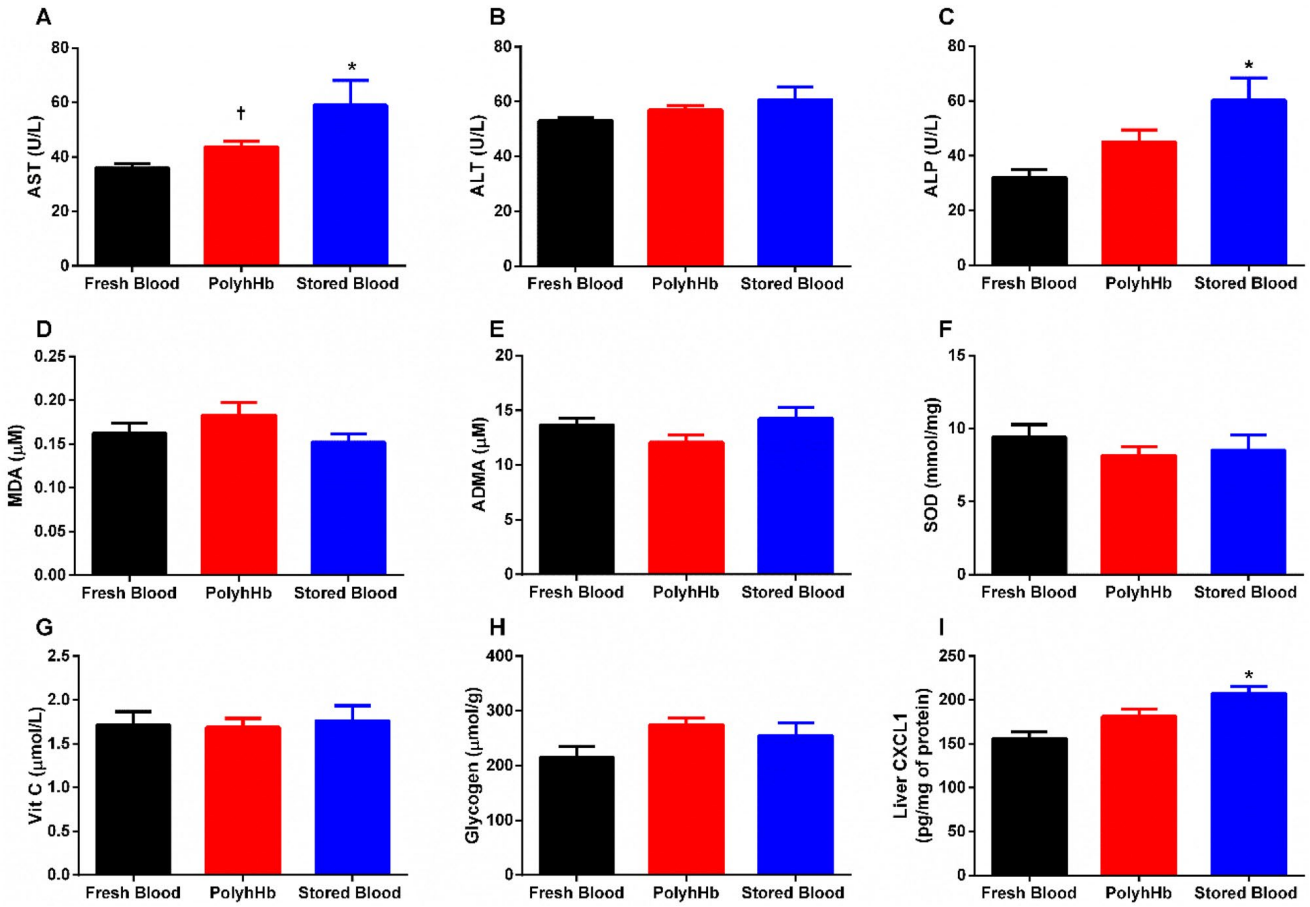
Markers of liver function after resuscitation. These results indicate that animals resuscitated with stored blood presented an increased risk for liver damage at 24 h after resuscitation, while resuscitation with PolyhHb do not seem to drastically impair liver enzymes. (**A**) Aspartate aminotransferase (AST); (**B**) Alanine aminotransferase (ALT); (**C**) Alkaline phosphatase (ALP); (**D**)  Malondialdehyde (MDA); (**E**) Asymmetric dimethylarginine (ADMA); (**F**) Superoxide dismutase (SOD); (**G**) Vitamin C (Vit C); (**H**) Glycogen; (**I**) Liver chemokine ligand 1 (CXCL-1). Measurements were taken 24 h after resuscitation. *, *p* < 0.05 versus Fresh Blood; †, *p* < 0.05 versus Stored Blood. Statistical comparisons were completed using a one way- ANOVA; Tukey’s multiple comparison test.

It has been extensively reported that transfusion of early generations of HBOCs can increase oxidative stress leading to organ damage, making it important to evaluate damage to vital organs upon transfusion^19–21^. In this study, we used a set of classical biomarkers to determine early kidney or liver injury in response to resuscitation with blood or PolyhHb. Biomarkers of liver damage are presented in Fig. 2 (panels A to I). Aspartate aminotransferase (AST) was statistically higher after resuscitation with SB (59.0 ± 9.1U/L) compared to FB (35.9 ± 1.6 U/L) (p < 0.01), but was not different from resuscitation with PolyhHb (43.7 ± 2.0 U/L) (p = 0.05). AST was not different between resuscitation with FB and PolyhHb. Alkaline phosphatase (ALP) was statistically higher after resuscitation with SB (60.3 ± 8.1 U/L) compared to FB (32.1 ± 2.9 U/L) (*p* < 0.01). Alanine aminotransferase (ALT) after resuscitation was not different between groups. Moreover, no differences were observed for malondialdehyde (MDA), asymmetric dimethylarginine (ADMA), superoxide dismutase (SOD), vitamin C (vit C), and glycogen between groups. The SB group presented statistically higher chemokine ligand-1 (CXCL1) (207.7 ± 8.0 pg/mg of protein), a liver inflammatory marker, compared to the FB group (155.9 ± 7.5 pg/mg of protein) (*p* < 0.01). Reperfusion with PolyhHb did not change liver CXCL1 compared to FB or SB groups.

**Figure 3:**
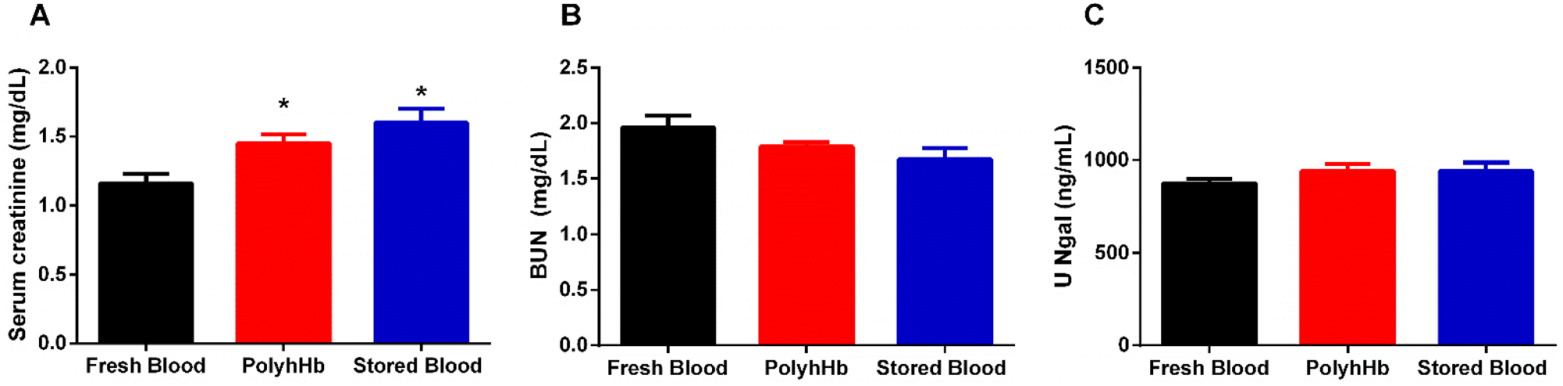
Markers of kidney function after resuscitation. Stored blood and PolyhHb groups indicate changes in kidney function compared to the fresh blood group, as observed by increased serum creatinine. However, the level of dysfunction could be transient since BUN and U Ngal, both kidney damage markers, did not change when compared to the fresh blood group. (**A**) Serum creatinine; (**B**) Blood urea nitrogen (BUN); (**C**) Urinary neutrophil gelatinase-associated lipocalin (U Ngal). Measurements were taken 24 h after resuscitation. *, *p* < 0.05 versus Fresh Blood. Statistical comparisons were completed using a one way—ANOVA; Tukey’s multiple comparison test.

Markers of kidney function after resuscitation are shown in Fig. 3. The SB group (1.6 ± 0.1 mg/dL, *p* < 0.01) and the PolyhHb group (1.5 ± 0.1 mg/dL, *p* = 0.03) showed statistically higher creatinine compared to the FB group (1.2 ± 0.1 mg/dL). Blood urea nitrogen (BUN) and urine neutrophil gelatinase-associated lipocalin (U Ngal) (classic markers of kidney injury) were not different between groups, suggesting the absence of kidney damage after resuscitation.

#### Heart

**Figure 4:**
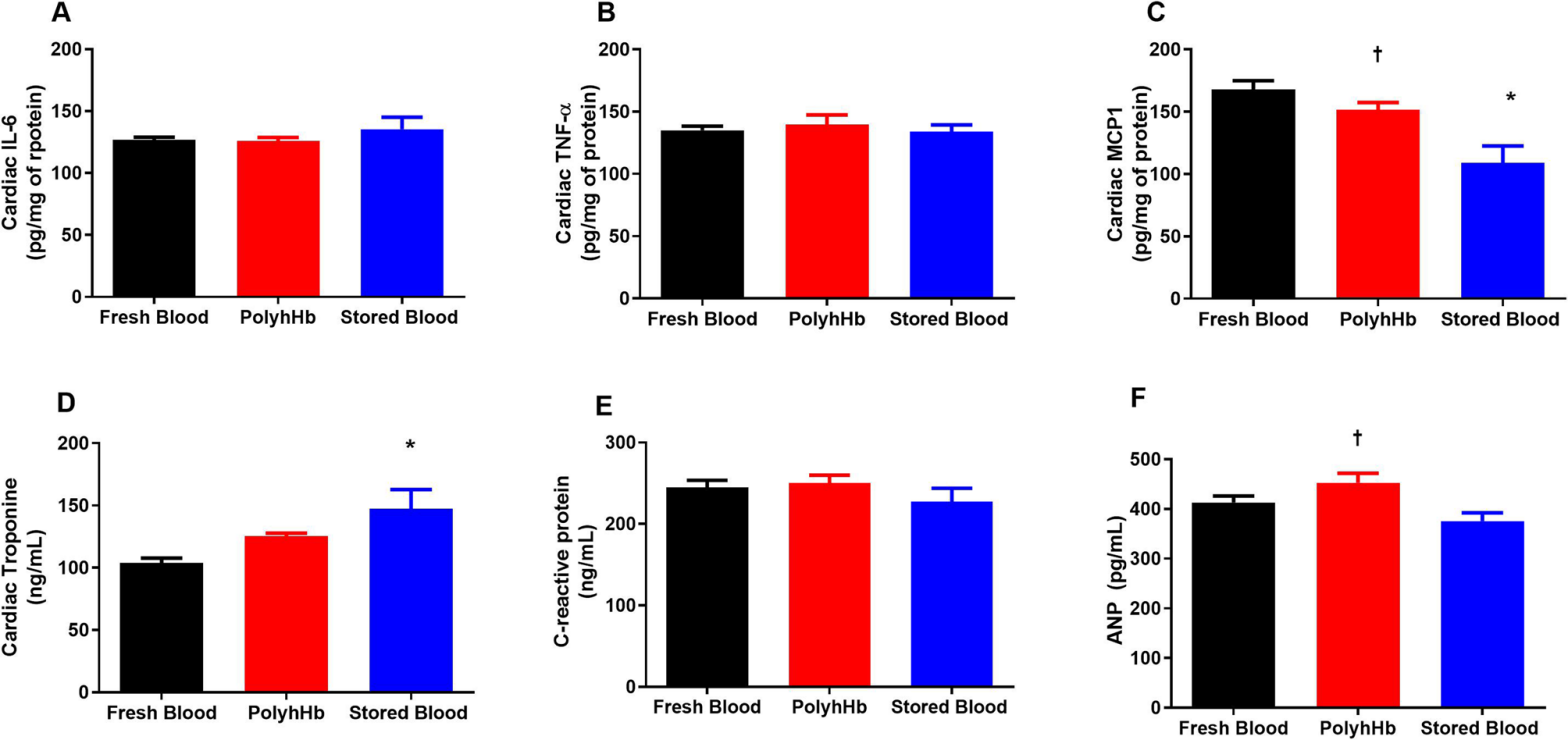
Markers of cardiac injury. Stored blood caused a greater degree of cardiac injury compared to fresh blood, since cardiac troponin increased for stored blood. However, resuscitation with stored blood or PolyhHb did not cause remarkable changes in cardiac inflammation. (**A**) Interleukin-6 (IL-6); (**B**) Tumor necrosis factor alpha (TNFα); (**C**) Monocyte chemoattractant protein-1 (MCP-1); (**D**) Troponin; **(E)** C-reactive protein; **(F) **Atrial natriuretic peptide (ANP). Measurements were taken 24 h after resuscitation. *, *p* < 0.05 vs. Fresh Blood; †, *p* < 0.05 versus Stored Blood. Statistical comparisons were completed using a one way—ANOVA; Tukey’s multiple comparison test.

Early generations of HBOCs were reported to elicit vasoconstriction, hypertension, and cardiovascular events^12,22^. Thus, our study aimed to track possible cardiac damage after resuscitation. Cardiac troponin, c-reactive protein, atrial natriuretic peptide (ANP), and inflammatory markers were evaluated to indirectly assess any cardiac damage (Fig. 4). No significant differences between groups were observed for heart inflammatory interleukin (IL) − 6 (FB: 126.7 ± 2.3; PolyhHb: 125.7 ± 3.1; SB: 135.5 ± 9.6 pg/mg of protein), and tumor necrosis factor-α (TNF-α) (FB: 135.1 ± 3.3; PolyhHb: 139.7 ± 7.7; SB: 134.1 ± 5.3 pg/mg of protein), however the SB group presented between 35 and 30% lower levels of monocyte chemoattractant protein-1 (MCP-1) in the heart compared to the FB (*p* < 0.01) and PolyhHb (*p* < 0.01) groups (FB: 167.9 ± 6.9; PolyhHb: 151.8 ± 5.6; SB: 109.1 ± 13.4 pg/mg of protein). Cardiac troponin was statistically higher (41%) for the SB group compared to the FB group (*p* < 0.01) (FB: 103.8 ± 4.0; PolyhHb: 125.3 ± 2.5; SB: 147.4 ± 15.4 ng/mg of protein), while c-reactive protein was no different between groups (FB: 245.2 ± 8.5; PolyhHb: 250.4 ± 9.4; SB: 227.6 ± 16.3 ng/mg of protein). The increased cardiac troponin level indicated cardiac damage caused by resuscitation with stored blood. Surprisingly, ANP levels were 17% lower in the SB group compared to the PolyhHb group (p < 0.01) (FB: 413.1 ± 12.9; PolyhHb: 452.6 ± 19.5; SB: 375.6 ± 16.8 ng/mg of protein).

#### Systemic and splenic inflammatory markers, and catecholamines

**Figure 5:**
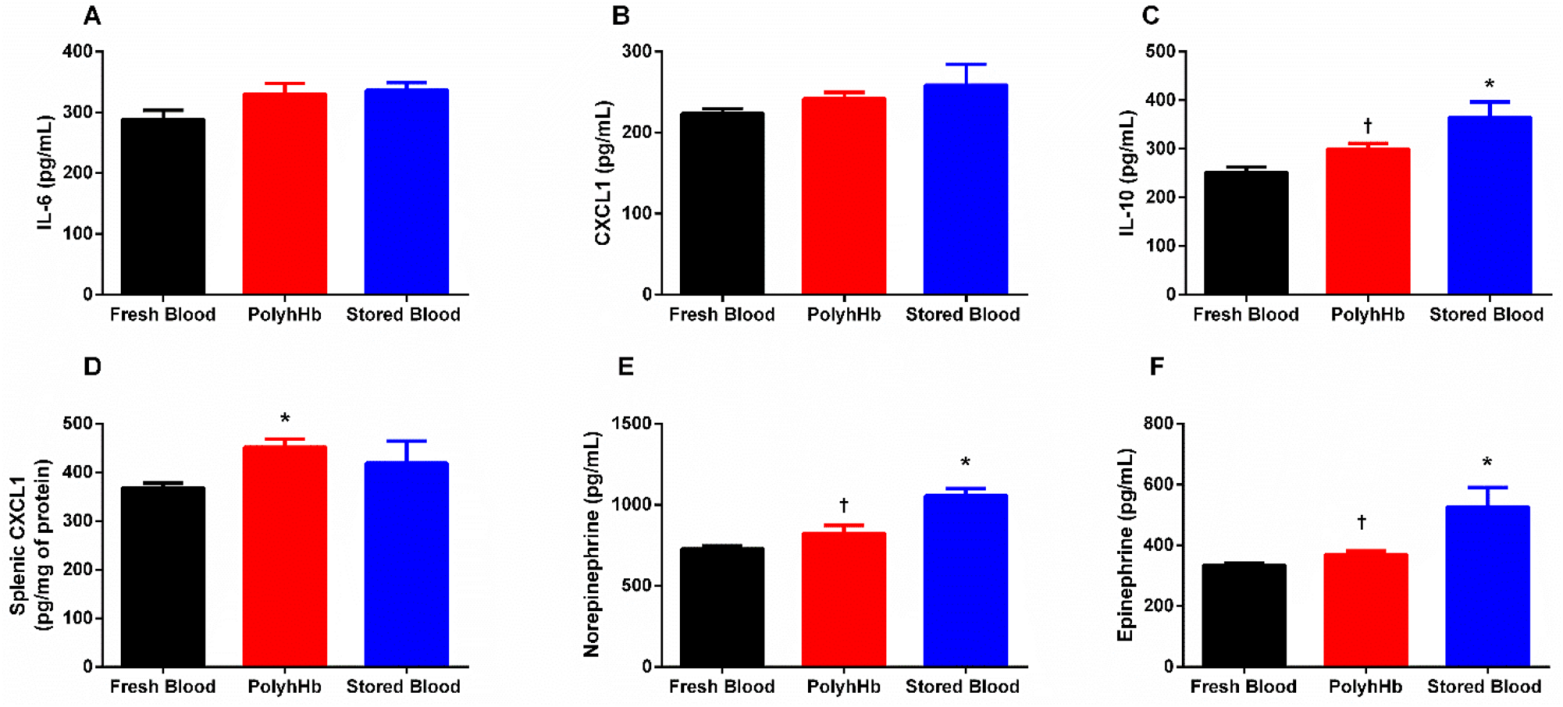
Systemic inflammation, splenic inflammation, and catecholamines. Neither PolyhHb or stored blood presented higher systemic inflammation compared to fresh blood (the gold standard for resuscitation). However, stored blood increased catecholamine levels compared to fresh blood and PolyhHb suggesting higher sympathetic activation after resuscitation. (**A**) Interleukin-6 (IL-6); (**B**) Chemokine ligand 1 (CXCL-1); (**C**) Interleukin-1(IL-10); (**D**) Splenic CXCL-1; (**E**) Norepinephrine; (**F**) Epinephrine. Measurements were taken 24 h after resuscitation. *, *p* < 0.05 versus Fresh Blood; †, *p* < 0.05 versus Stored Blood. Statistical comparisons were completed using a one way—ANOVA; Tukey’s multiple comparison test.

Inflammatory responses are expected from blood transfusions, and are exacerbated by transfusion of stored blood and, potentially by PolyhHb^20,23,24^. Plasma and spleen inflammatory markers are presented in Fig. 5. Plasma IL-6 (FB: 288.6 ± 15.3; PolyhHb: 330.2 ± 17.7; SB: 336.1 ± 12.7 pg/mL) and CXCL1 (FB: 223.7 ± 6.0; PolyhHb: 242.1 ± 7.6; SB: 258.6 ± 25.7 pg/mL), two inflammatory markers, were not statiscally different in the SB and PolyhHb groups compared to the FB group suggesting there was no systemic inflammation. In contrast, the SB group presented 45% higher levels of IL-10 (an anti-inflammatory marker) compared to the FB group (*p* < 0.01), and 22% higher compared to the PolyhHb group (*p* = 0.04) (FB: 251.7 ± 11.3; PolyhHb: 299.8 ± 11.3; SB: 365.0 ± 31.62 pg/mL). Moreover, splenic CXCL1 was higher only in the PolyhHb group by 23% compared to the FB group (p = 0.04), and was no different compared to the SB group (FB: 368.3 ± 9.7; PolyhHb: 452.0 ± 17.0; SB: 418.8 ± 45.8 pg/mg of protein).

Catecholamines are autonomic nervous system biomarkers and can predict cardiovascular risk^25^. At 24 h after resuscitation, the SB group had sympathetic hyperactivation, as suggested by higher norepinephrine compared to the FB and PolyhHb groups (p < 0.01 and p < 0.01) (FB: 728.3 ± 18.9; PolyhHb: 824.7 ± 50.1; SB: 1055 ± 44.8 pg/mL). Similarly, epinephrine was higher after resuscitation in the SB group compared to the FB and PolyhHb groups (p < 0.01 and p < 0.01) (FB: 333.6 ± 7.0; PolyhHb: 370.1 ± 11.8; SB: 525.6 ± 64.5 pg/mL).

#### Iron metabolism

Oxidative damage caused by ferryl Hb is one of the major adverse chemical reactions associated with prior generations of HBOCs^26,27^. In the present study, the enzyme ferritin was measured in all organs as well as total bilirubin, in an effort to understand iron metabolism and track possible adverse effects caused by either blood or PolyhHb. Serum, spleen, liver, heart ferritin and bilirubin after resuscitation are presented in Table 2. Serum ferritin, spleen ferritin, and heart ferritin was lower in the SB group compared to the FB (*p* < 0.01) group and the PolyhHb group (*p* < 0.01). Liver ferritin was lower in the SB group compared only to the PolyhHb group (*p* = 0.04). Total bilirubin was lower in the fresh blood group compared to the PolyhHb group (*p* = 0.04) and the stored blood group (*p* < 0.01).

**Table 2:** Iron metabolism.

	Fresh blood (FB)	PolyhHb	Stored blood (SB)
Serum ferritin (µg/L)	445.4 ± 19.5	417.7 ± 19.3†	306.3 ± 9.9*
Spleen ferritin (µg/g)	595.0 ± 26.5	534. 5 ± 29.9†	397.1 ± 23.9*
Heart ferritin (µg/g)	710.3 ± 24.7	617.1 ± 30.6†	375.1 ± 22.5*
Liver ferritin(µg/g)	339.7 ± 11.5	333.7 ± 24.2†	251.2 ± 5.6
Total bilirubin (mg/dL)	5.0 ± 0.3	7.3 ± 0.4*	9.2 ± 2.8*

Imbalanced iron metabolism was observed in the group resuscitated with stored blood, suggesting a reduced capacity for processing the excess of iron present in the circulation after resuscitation, and this imbalance could be associated with the organ injury observed in this group. Data was presented as the mean ± SE. *p < 0.05 versus Fresh Blood, †*p* < 0.05 versus Stored Blood. Statistical comparisons were completed using a one way—ANOVA; Tukey’s multiple comparison test.

## Discussion

The main finding in the present study was that high MW PolyhHb can restore systemic hemodynamics after HS in guinea pigs that was comparable to blood. Additionally, our results indicated that resuscitation with high MW PolyhHb displayed insignificant toxicity towards the heart, liver, spleen, and kidney based on classical markers of organ function and inflammation. These results are promising and exciting as they demonstrate a positive safety profile for high MW PolyhHb that is comparable to blood in an animal species with similar antioxidant capacity to humans^16^. The results support the hypothesis that high MW PolyhHb can restore hemodynamics, and causes minimal toxicity in this clinically relevant animal model.

PolyhHb efficiently recovered hemodynamics after resuscitation from HS compared to fresh blood and stored blood. All groups recovered MAP equally after resuscitation with no differences in HR. We showed similar results with a high MW bovine polymerized Hb (PolybHb) in a previous study^28^. This previous study was completed in rats resuscitated with PolybHb, and showed that PolybHb restored both the O_2_ carrying capacity and MAP comparable to blood^28^. Similar outcomes have been previously reported for other HBOCs, such as PEGylated albumin-heme^29^, PolybHb^30^, polyethylene glycol-modified human Hb^31^, Hb vesicles^32^, and polymerized human placental Hb^33^. Taken together our results and those from the literature provide strong evidence that HBOCs can be a suitable option to treat trauma associated blood loss when blood is not available. However, our results are especially exciting, because we tested the PolyhHb in a guinea pig model, and as mentioned before these animals are a more relevant model to evaluate HBOCs safety and efficacy due to their similar antioxidant status to humans^16^.

Other HBOCs formulations have been evaluated in HS models, e.g. Li et al. demonstrated that polymerized human placental Hb improved hemodynamic recovery after HS but provided little protective effect against cardiac dysfunction^33^. The authors discussed that the reason for the negative cardiac effects observed in their study stemmed from the presence of unpolymerized Hb in solution, which caused nitric oxide (NO) scavenging and vasoconstriction. They also hypothesized that increasing the structural stability of the polymerized Hb to reduce the concentration of cell-free Hb could be a possible solution to resolve this issue. Moreover, the solution tested in that study included polymerized Hb molecules with low MW (64–600 kDa)^33^, which our previous studies have demonstrated that causes increased blood pressure, vasoconstriction, and toxicity^34,35^. In the present study, our next generation high MW PolyhHb appears to be superior to stored blood, in terms of cardiac injury, as suggested by cardiac markers. In fact, the increased cardiac troponin of the stored blood group, a classical marker of cardiac dysfunction, suggests that stored blood might increase cardiovascular risks when compared to PolyhHb. These results demonstrate that high MW PolyhHb does not elicit cardiac side-effects, corroborating our previous publication where we demonstrated that increasing the MW of the polymerized Hb decreases its’ toxicity^14^.

During HS, the reduced blood flow limits O_2_ delivery and tissues become hypoxic and are forced to switch to anaerobic metabolism, which increases lactate production. Therefore, lactate is a good marker of metabolic recovery from HS given its strong relationship to inadequate tissue oxygenation and morbidity and mortality^36^. In this study, all groups increased lactate during HS, but PolyhHb was the only group that did not return lactate to baseline levels after 2 h post-resuscitation, besides not being statistically significant, the p value compared to fresh blood group was p = 0.06. The increase in lactate could be a result of tissue hypoperfusion, although other markers do not seem to support the idea of reduced blood flow after resuscitation with PolyhHb, moreover, the pH was normal and similar for all groups, suggesting a lack of metabolic acidosis. There is no indication that PolyhHb impairs the clearance of lactate, as liver enzymes were normal for animals resuscitated with PolyhHb. Despite the acute increase at 2 h after resuscitation, at 24 h after resuscitation lactate levels were restored and pH was also within the normal range for PolyhHb resuscitated animals suggesting that the excess lactate at 2 h was cleared out by the liver. Our group has tested HBOCs in diverse animal models, however the experimental design is different between these studies, thus a comprehensive comparison of HBOC efficacy to restore lactate levels after resuscitation from hemorrhage using similar experimental models is necessary, and although we have evaluated PolyhHb in rats and guinea pigs, the protocol and species physiological differences challenge the analysis of the effects of PolyhHb on the recovery of lactate after resuscitation.

All groups presented similar tHb at 2 h after resuscitation, but after 24 h the PolyhHb group showed a lower level of tHb compared to the fresh blood and stored blood groups. The main reason for the reduction in tHb for the PolyhHb group after 24 h is its’ clearance from the circulation by the liver and spleen (circulatory half-life estimated to be between 11 and13 h)^13^. More specifically, PolyhHb is trapped by the reticuloendothelial system and metabolized similarly to cell-free Hb^14^. The reduced circulatory half-life makes it challenging to preserve O_2_ carrying capacity beyond 12 h, and we are working on developing approaches to increase the circulatory half-life of PolyhHb to further take advantage of its’ O_2_ carrying capacity for longer periods of time. Despite the lower tHb 24 h after resuscitation, PolyhHb provided sufficient O_2_ carrying capacity to restore hemodynamics in the present model, as no signs of anaerobic metabolism were noted in the PolyhHb group compared to the fresh blood and stored blood groups. Moreover, the Hct was lower for the PolyhHb group compared to the other groups, because of the lack of RBCs in the PolyhHb formulation.

In addition to its’ O_2_ carrying capacity, high MW PolyhHb has many other ideal biophysical characteristics that enable restoration of hemodynamics after HS such as its’ high viscosity to restore blood rheology and endothelial mechano-transduction, and high colloid osmotic pressure (COP) to retain intravascular fluids and prevent PolyhHb extravasation into the tissue space^37,38^. Our previous studies showed that O_2_ delivery, viscosity, and COP are very important factors to consider for recovering blood pressure, cardiac function, and blood flow after resuscitation from HS^37^. Moreover, it has been discussed in the literature that the O_2_ carrying capacity of the resuscitation fluid is one of the main characteristics that enables effective resuscitation^33^. A previous study demonstrated that resuscitating animals with solutions with increased O_2_ carrying capacity increased survival from 40 to 80%^33^. The blood pressure and hematological parameters results presented herein suggest that these optimal biophysical characteristics are playing an important role in restoring hemodynamics and avoiding tissue toxicity in the treatment of HS in the guinea pig model.

Interestingly, only stored blood showed an increase in AST, ALP, and liver CXCL1 compared to fresh blood, suggesting that stored blood increases the risk of liver damage. The same effect was not observed in the PolyhHb group, suggesting a lack of liver toxicity by PolyhHb. These results are consistent with a previous study from our group, where we demonstrated that stored blood exhibited higher liver toxicity in rats compared to a high MW PolybHb^39^. Furthermore, You et al*.* demonstrated that isovolumic hemodilution with glutaraldehyde polymerized human placental Hb attenuated rat liver ischemia/reperfusion injury, reinforcing the notion that PolyhHb is a good resuscitation fluid that can preserve liver function in trauma models^40^.

Serum creatinine, BUN, and urine Ngal were measured to assess the resuscitation solution effects on kidney function. Increased serum creatine suggests that both stored blood and PolyhHb cause kidney dysfunction, while the lack of changes in BUN and UNgal levels demonstrates that PolyhHb did not cause further kidney injury. Changes in kidney function are typically acute and could be transient, as previously demonstrated by Zhang et al*.*^41^. To better clarify the extent of the change in kidney function, additional experiments will be necessary to assess renal function after 48 h. Acute kidney injury (AKI) after RBC transfusion is usually associated with excess iron in the circulation and if ferritin is not present to remove the excess of iron, it can cause even worse clinical outcomes. Baek et al*.* demonstrated that administration of a ferroportin inhibitor to switch compartmentalization of iron from the plasma to the spleen and liver, prevents post-transfusion iron accumulation, oxidative stress, and AKI^42^. Unlike the published study, we elected to use whole blood instead of RBCs, but we believe that potential iron toxicity still remains as mechanism of iron toxicity. It is important to highlight that our results demonstrate the renal safety of PolyhHb compared to fresh and stored blood, the gold standard treatments for HS. Since potentially all groups in this study were subjected to renal ischemia during the HS protocol, all groups might have some degree of kidney damage. Hence, we expect to evaluate kidney safety of PolyhHb in models more appropriate to answer this question in future studies.

Ferritin and total bilirubin were measured as markers of iron metabolism. Free iron is a labile toxin that contributes to the generation of reactive oxygen species, lipid peroxides, and cellular injury. Ferritin is an enzyme that helps with the storage of iron, and it is rapidly expressed to eliminate the redox active form of free iron and as a result avoids tissue damage from oxidative stress^43^ Stored blood presented lower ferritin levels in all tissues, while PolyhHb ferritin levels remained similar to fresh blood. It is interesting to highlight that fresh blood and PolyhHb had higher levels of ferritin compared to normal levels in a Sham animal (unpublished data). In this scenario, the inability of animals resuscitated with stored blood to increase ferritin levels could be one of the reasons that we observed increased tissue damage in this group, since the excess iron that is not metabolized by ferritin causes higher redox activity and oxidative stress.

Increased sympathetic nervous system activity is one possible mechanism for the hypertension observed in previous generations of HBOCs^25^. In the present study, we measured catecholamine levels in order to trace all possible side-effects caused by PolyhHb. The increased catecholamines in the SB group suggests that resuscitation with stored blood caused sympathetic nervous system activation, and could explain the cardiac injury observed in the same group. These results are in agreement with the literature, since it is known that stored blood and stored RBCs are associated with adverse events and increased mortality 24 h post transfusion^3,4^.

Although HBOCs have been extensively studied, the inflammatory response resulting from HBOC transfusion remains unclear and controversial in the literature. For example, Ortegon et al*.* observed that HBOC-201 had immune-activating potential^44^, while Zhu et al*.* demonstrated that a porcine polymerized Hb did not result in a significant inflammatory response^24^. These conflicting results could be explained by the different formulations of solutions or the time after transfusion that the inflammatory pathway was evaluated. It is important to develop a resuscitation solution with minimal effects on inflammatory pathways, especially since it is well known that trauma leads to innate inflammatory activation^45,46^. We addressed this concern by evaluating IL-6, IL-10, and CXCL-1. We did not observe increased levels of systemic inflammatory markers in the stored blood group and PolyhHb group compared to the fresh blood group, but the stored blood group increased the anti-inflammatory marker IL-10. Our previous publication comparing stored blood with high MW PolybHb in rats showed an acute increase in inflammatory markers in both PolybHb and stored blood compared to fresh blood^39^. Our results in guinea pigs are 24 h post-resuscitation, suggesting that the increased inflammation observed in the rat model could be acute and transient due to the 2-h observation window in that study. Moreover, since guinea pigs are a more clinically relevant model, the lack of inflammatory response in the group resuscitated with PolyhHb is very promising. Further investigations are still necessary, including measurement of other markers of inflammation, specifically to explain the reason for the increased IL-10 in the stored blood group. Based on the anti-inflammatory role of IL-10^47^, our opinion is that the stored blood group increased IL-10 as a protective response to other inflammatory mediators resulting from the resuscitation with stored blood or was present in the stored blood itself, an effect not observed for the PolyhHb group, but worthy of future assessment.

Whole blood was chosen in the present study, due to its long history as a resuscitation fluid in military medicine^48^, with several studies showing that whole blood is the most optimal resuscitation fluid for treating trauma patients suffering from hemorrhage^48–50^. In this study, autologous shed blood was used as the fresh blood group, which could be impacting the results for this group, because inflammatory mediators and cytokines are released during the hemorrhage and the reinfusion of the shed blood during resuscitation can impact the outcome of the resuscitation. It is important to highlight that the use of whole blood in the fresh and stored groups in this study is a limitation, as apoptotic platelets and neutrophils might release extracellular vesicles, cytokines, and bioactive lipids that may not reflect the changes associated with storage of leukoreduced RBCs^51^. A further limitation is that the stored blood was collected in citrate phosphate double dextrose (CP2D) and Additive Solution 3 (AS-3), but the fresh blood was not, this could also interfere with the results presented herein, and further studies are necessary to ensure that the observed side-effects are due to the ex vivo storage time and not due to the blood preparation. The guinea pig RBC in vivo circulatory lifetime is not comparable to the human RBC circulatory lifetime, and it is expected that the storage limits are completely different between species. However, animal models are often used as a translational tool to understand the mechanisms behind the RBC or whole blood storage lesion. Our rationale for storing guinea pig blood for 2 weeks is based on the fact that the normal circulatory lifetime of human RBCs is 120 days, and human whole blood can be stored for 21 days (nearly 18% of the normal RBC circulatory lifetime), and the circulatory lifetime of guinea pig RBCs is 80 days and 18% of their circulatory lifetime is 14 days (2 weeks). In addition, Bertolone et al. demonstrated that the hemolysis at the end of ex vivo storage for humans is equal to the hemolysis of guinea pig stored blood after 2 weeks^52^. On the other hand, Baek et al. showed that a 28-day old guinea pig RBC unit was equivalent to a 42-day old human RBC unit^49^, thus highlighting the need for further studies to clarify the adequate time of ex vivo storage to compare guinea pig studies to human studies. Finally, the immune effects originating from white blood cells and platelets are very relevant towards the evaluation of HBOCs. In future studies, we intend to explore the impact of platelet-sparing and leukodepletion on whole blood function relative to PolyhHb. We expect to use an isovolumic transfusion model since the HS model's effects complicate the interpretation of ischemic, hypoxic, and inflammatory changes resulting from administration of the transfusion solution.

## Conclusion

These results suggest that high MW PolyhHb was as effective in resuscitating guinea pigs from HS as fresh blood. Stored blood increased the risk of liver and cardiac damage along with sympathetic hyperactivation, side-effects that were not observed with PolyhHb. Additional experiments should be done to ensure PolyhHb’s safety before moving forward to clinical trials, such as measurements of oxidative stress, markers of iron tissue accumulation, and other inflammatory pathways that could be activated in this model. Nevertheless, these results are promising and encourage further evaluation of this next generation PolyhHb as an alternative to blood when blood is not available.

## Methods

### Animal preparation

Animal handling and care followed the National Institutes of Health Guide for the Care and Use of Laboratory Animals, and the University of California San Diego Institutional Animal Care and Use Committee approved the experimental protocol. All methods were carried out in accordance with the ARRIVE guidelines (Animal Research: Reporting of In Vivo Experiments). Guinea pigs weighing between 300–400 g were used. Animals were placed on a heating pad to maintain their core body temperature at 37 °C for any procedures or experimental protocols that were performed under anesthesia. Guinea pigs were anesthetized with isoflurane (Drägerwerk AG, Lübeck, Germany) in compressed room air (flow rate 1.0 LPM) slowly, by increasing the isoflurane 0.4% every 3 min until a surgical depth of anesthesia was achieved, typically 3%. This ensured that the animals did not stop breathing due to airway irritation by isoflurane and prevented variations in heart rate (HR). Animals were instrumented with catheters in the right carotid artery and left jugular vein, which were exteriorized dorsally.

### Blood collection and preparation

**Fresh blood:** Blood withdrawn from the animal during hemorrhage (autologous blood), and kept at room temperature until it was reinfused for resuscitation. **Stored blood:** A total of 4 male guinea pigs were anesthetized with isoflurane (5%). Blood was collected via cardiac puncture into citrate phosphate double dextrose (CP2D), which was taken from an Additive Solution 3 (AS-3) blood preparation kit (Haemonetics Corporation, Braintree, MA). Donor blood was then pooled, and the CP2D concentration was adjusted to 14%. AS-3 (22%/whole blood volume) was then added, and the blood was mixed gently by inverting the bag for 1 min. Lastly, the whole blood was stored at 4 °C for 2 weeks. The day of the experiments, the stored whole blood was centrifuged at 1,500 g for 7 min, and some of plasma was removed to adjust the hematocrit (HCT) to 40% to match the Hct of the fresh blood group. Stored blood was warmed before infusion during resuscitation.

### Polymerized human hemoglobin (PolyhHb)

PolyhHb was synthesized in the low O_2_ affinity quaternary tense state (T-state) at a 30:1 molar ratio of glutaraldehyde to human Hb, filtered through a 0.2 µm hollow fiber filter, and then subjected to 8–9 cycles of diafiltration on a 500 kDa hollow fiber filter. This resulted in a PolyhHb solution containing only polymerized Hb molecules with molecular weight (MW) greater than 500 kDa but less than 0.2 µm in size. PolyhHb was produced and characterized at The Ohio State University and shipped overnight frozen to UC San Diego where it was stored at − 80 °C until use. The preparation and characterization of high MW T-state PolyhHb has been previously described in the literature^38,53^.

### Hemorrhagic shock (HS)

HS was induced by withdrawing 40% of the blood volume (BV) from the carotid artery (BV estimated as 7.5% of body weight), and the hypovolemic state was maintained for 50 min. After being subjected to 50 min of HS, guinea pigs were divided into three study groups, and 25% of their BV was reinfused with fresh blood, stored blood or PolyhHb at a Hb concentration of 10 g/dL (PolyhHb) (n = 6 animals/group).

### Hemodynamic and hematological measurements

The arterial cannula was connected to a pressure transducer and recording system (MP150, Biopac, Santa Barbara, CA), and blood pressure signals were recorded, along with mean arterial pressure (MAP), and heart rate (HR). The Hct was measured from centrifuged arterial blood samples taken in heparinized capillary tubes. Arterial blood was collected in heparinized glass capillaries (50 μL) and immediately analyzed for pO_2_, pCO_2_, pH, electrolytes, lactate, and total Hb content (ABL90; Radiometer America, Brea, CA). All these measurements were taken at baseline, HS, 15 min after resuscitation, and animals were allowed to recover from anesthesia. Additionally, measurements were taken without anesthesia at 2 h and 24 h after resuscitation.

### Harvesting tissues

Guinea pigs were anesthetized with isoflurane 5%, and 10 mL of blood was collected from the implanted arterial catheter and centrifuged (3000 RPM for 7 min) to separate the plasma. Guinea pigs were euthanized with Fatal Plus (sodium pentobarbital, 300 mg/kg), and urine collected, while the following organs were harvested: kidneys, liver, spleen, and heart. Markers of inflammation, organ function, and organ injury were evaluated. These analyses were performed by the UC San Diego Histology Core via ELISA and flow cytometric analysis of tissue homogenates and plasma. The kits and methods used for these analyses are described in Supplemental Table S1.

### Statistical analysis

All values are expressed as the mean ± SE. Data between groups were analyzed using two-way analysis of variance (ANOVA), with Tukey's post hoc test when necessary for the parameters over time. One-way ANOVA was used for the tissue measurement taken only at 24 h. All statistics were calculated using GraphPad Prism 6 (GraphPad Software, Inc., San Diego, CA). The specific Tukey's post hoc multiple comparisons test p-values were reported only if differences were significant (*p* < 0.05) between groups.

## Supplementary Information

Supplementary Information.

## References

[1] Eastridge BJ, Holcomb JB, Shackelford S (2019). Outcomes of traumatic hemorrhagic shock and the epidemiology of preventable death from injury. Transfusion.

[2] Edwards TH, Hoareau GL (2020). Fluids of the future. Front. Vet. Sci..

[3] Jones AR, Patel RP, Marques MB, Donnelly JP, Griffin RL, Pittet JF (2019). Older blood is associated with increased mortality and adverse events in massively transfused trauma patients: Secondary analysis of the PROPPR trial. Ann. Emerg. Med..

[4] Oh JY, Marques MB, Xu X, Li J, Genschmer K, Gaggar A (2020). Damage to red blood cells during whole blood storage. J. Trauma Acute Care Surg..

[5] Chung KW, Basavaraju SV, Mu Y, van Santen KL, Haass KA, Henry R (2016). Declining blood collection and utilization in the United States. Transfusion.

[6] Meledeo MA, Peltier GC, McIntosh CS, Bynum JA, Cap AP (2019). Optimizing whole blood storage: Hemostatic function of 35-day stored product in CPD, CP2D, and CPDA-1 anticoagulants. Transfusion.

[7] Zimrin AB, Hess JR (2009). Current issues relating to the transfusion of stored red blood cells. Vox Sang..

[8] Hess JR (2010). Red cell changes during storage. Transfus. Apher. Sci. Off. J. World Apher. Assoc. Off. J. Eur. Soc. Haemapheresis.

[9] Hess JR, Brohi K, Dutton RP, Hauser CJ, Holcomb JB, Kluger Y (2008). The coagulopathy of trauma: A review of mechanisms. J. Trauma.

[10] Hooper N, Armstrong TJ (2021). Hemorrhagic shock.

[11] Cooper ES, Silverstein DC (2021). Fluid therapy and the microcirculation in health and critical illness. Front. Vet. Sci..

[12] Moore EE, Moore FA, Fabian TC, Bernard AC, Fulda GJ, Hoyt DB (2009). Human polymerized hemoglobin for the treatment of hemorrhagic shock when blood is unavailable: The USA multicenter trial. J. Am. Coll. Surg..

[13] Muller CR, Williams AT, Munoz CJ, Eaker AM, Breton AN, Palmer AF (2021). Safety profile of high molecular weight polymerized hemoglobins. Transfusion.

[14] Williams AT, Muller CR, Eaker AM, Belcher DA, Bolden-Rush C, Palmer AF (2020). Polymerized hemoglobin with increased molecular size reduces toxicity in healthy guinea pigs. ACS Appl. Bio Mater..

[15] Muller CR, Courelli V, Lucas A, Williams AT, Li JB, Dos Santos F (2021). Resuscitation from hemorrhagic shock after traumatic brain injury with polymerized hemoglobin. Sci. Rep..

[16] Buehler PW, D'Agnillo F, Hoffman V, Alayash AI (2007). Effects of endogenous ascorbate on oxidation, oxygenation, and toxicokinetics of cell-free modified hemoglobin after exchange transfusion in rat and guinea pig. J. Pharmacol. Exp. Ther..

[17] Chatterjee IB (1973). Evolution and the biosynthesis of ascorbic acid. Science.

[18] Dutton RP (2007). Current concepts in hemorrhagic shock. Anesthesiol. Clin..

[19] Hod EA, Spitalnik SL (2011). Harmful effects of transfusion of older stored red blood cells: Iron and inflammation. Transfusion.

[20] Hod EA, Zhang N, Sokol SA, Wojczyk BS, Francis RO, Ansaldi D (2010). Transfusion of red blood cells after prolonged storage produces harmful effects that are mediated by iron and inflammation. Blood.

[21] Natanson C, Kern SJ, Lurie P, Banks SM, Wolfe SM (2008). Cell-free hemoglobin-based blood substitutes and risk of myocardial infarction and death—A meta-analysis. Jama-J. Am. Med. Assoc..

[22] Burhop K, Gordon D, Estep T (2004). Review of hemoglobin-induced myocardial lesions. Artif. Cell Blood Sub..

[23] Escobar GA, Cheng AM, Moore EE, Johnson JL, Tannahill C, Baker HV (2007). Stored packed red blood cell transfusion up-regulates inflammatory gene expression in circulating leukocytes. Ann. Surg..

[24] Zhu HL, Yan KP, Dang XD, Huang H, Chen EF, Chen B (2011). Immune safety evaluation of polymerized porcine hemoglobin (pPolyHb): A potential red blood cell substitute. Artif. Cell Blood Sub..

[25] Esler M, Kaye D (1998). Increased sympathetic nervous system activity and its therapeutic reduction in arterial hypertension, portal hypertension and heart failure. J. Autonom. Nerv. Syst..

[26] Alayash AI (2014). Blood substitutes: Why haven't we been more successful?. Trends Biotechnol..

[27] Huo SS, Lei XF, He D, Zhang H, Yang ZP, Mu WH (2021). Ferrous hemoglobin and hemoglobin-based oxygen carriers acting as a peroxidase can inhibit oxidative damage to endothelial cells caused by hydrogen peroxide. Artif. Organs.

[28] Ao-Ieong ES, Williams A, Jani V, Cabrales P (2017). Cardiac function during resuscitation from hemorrhagic shock with polymerized bovine hemoglobin-based oxygen therapeutic. Artif. Cells Nanomed. Biotechnol..

[29] Huang Y, Komatsu T, Yamamoto H, Horinouchi H, Kobayashi K, Tsuchida E (2006). PEGylated albumin-heme as an oxygen-carrying plasma expander: Exchange transfusion into acute anemia rat model. Biomaterials.

[30] Cabrales P, Tsai AG, Intaglietta M (2009). Polymerized bovine hemoglobin can improve small-volume resuscitation from hemorrhagic shock in hamsters. Shock.

[31] Wettstein R, Tsai AG, Erni D, Winslow RM, Intaglietta M (2003). Resuscitation with polyethylene glycol-modified human hemoglobin improves microcirculatory blood flow and tissue oxygenation after hemorrhagic shock in awake hamsters. Crit. Care Med..

[32] Sakai H, Horinouchi H, Masada Y, Takeoka S, Ikeda E, Takaori M (2004). Metabolism of hemoglobin-vesicles (artificial oxygen carriers) and their influence on organ functions in a rat model. Biomaterials.

[33] Li Y, Yan D, Hao S, Li S, Zhou W, Wang H (2015). Polymerized human placenta hemoglobin improves resuscitative efficacy of hydroxyethyl starch in a rat model of hemorrhagic shock. Artif. Cells, Nanomed. Biotechnol..

[34] Cabrales P, Sun G, Zhou Y, Harris DR, Tsai AG, Intaglietta M (2009). (1985) Effects of the molecular mass of tense-state polymerized bovine hemoglobin on blood pressure and vasoconstriction. J. Appl. Physiol..

[35] Baek JH, Zhou YP, Harris DR, Schaer DJ, Palmer AF, Buehler PW (2012). Down selection of polymerized bovine hemoglobins for use as oxygen releasing therapeutics in a guinea pig model. Toxicol. Sci..

[36] Levitt DG, Levitt JE, Levitt MD (2020). Quantitative assessment of blood lactate in shock: Measure of hypoxia or beneficial energy source. Biomed. Res. Int..

[37] Williams AT, Lucas A, Muller CR, Bolden-Rush C, Palmer AF, Cabrales P (2020). (1985) Balance between oxygen transport and blood rheology during resuscitation from hemorrhagic shock with polymerized bovine hemoglobin. J. Appl. Physiol..

[38] Cuddington C, Moses S, Belcher D, Ramesh N, Palmer A (2020). Next-generation polymerized human hemoglobins in hepatic bioreactor simulations. Biotechnol. Prog..

[39] Williams AT, Lucas A, Muller CR, Munoz C, Bolden-Rush C, Palmer AF (2020). Resuscitation from hemorrhagic shock with fresh and stored blood and polymerized hemoglobin. Shock.

[40] You Z, Li Q, Li B, Yang CM, Liu J, Li T (2014). Isovolemic hemodilution with glutaraldehyde-polymerized human placenta hemoglobin (PolyPHb) attenuated rat liver ischemia/reperfusion injury. Artif. Cell Nanomed. B..

[41] Zhang XY, Williams MC, Rentsendorj O, D'Agnillo F (2018). Reversible renal glomerular dysfunction in guinea pigs exposed to glutaraldehyde-polymerized cell-free hemoglobin. Toxicology.

[42] Baek JH, Shin HKH, Gao Y, Buehler PW (2020). Ferroportin inhibition attenuates plasma iron, oxidant stress, and renal injury following red blood cell transfusion in guinea pigs. Transfusion.

[43] Gall T, Balla G, Balla J (2019). Heme, Heme oxygenase, and endoplasmic reticulum stress-a new insight into the pathophysiology of vascular diseases. Int. J. Mol. Sci..

[44] Ortegon DP, Dixon PS, Crow KK, Mueller DL, Kerby JD (2003) The effect of the bovine hemoglobin oxygen therapeutic HBOC-201 on human neutrophil activation in vitro. The Journal of trauma.;55 (4):755–60; discussion 60–1. Epub 2003/10/21. 10.1097/01.TA.0000085722.52921.6D 14566134

[45] Fink MP (2002). Role of reactive oxygen and nitrogen species in acute respiratory distress syndrome. Curr. Opin. Crit. Care.

[46] Maier B, Lefering R, Lehnert M, Laurer HL, Steudel WI, Neugebauer EA (2007). Early versus late onset of multiple organ failure is associated with differing patterns of plasma cytokine biomarker expression and outcome after severe trauma. Shock.

[47] Iyer SS, Cheng G (2012). Role of interleukin 10 transcriptional regulation in inflammation and autoimmune disease. Crit. Rev. Immunol..

[48] Vanderspurt CK, Spinella PC, Cap AP, Hill R, Matthews SA, Corley JB (2019). The use of whole blood in US military operations in Iraq, Syria, and Afghanistan since the introduction of low-titer type O whole blood: Feasibility, acceptability, challenges. Transfusion.

[49] Nessen SC, Eastridge BJ, Cronk D, Craig RM, Berseus O, Ellison R (2013). Fresh whole blood use by forward surgical teams in Afghanistan is associated with improved survival compared to component therapy without platelets. Transfusion.

[50] Spinella PC, Perkins JG, Grathwohl KW, Beekley AC, Holcomb JB (2009). Warm fresh whole blood is independently associated with improved survival for patients with combat-related traumatic injuries. J. Trauma.

[51] Sharma RR, Marwaha N (2010). Leukoreduced blood components: Advantages and strategies for its implementation in developing countries. Asian J. Transfus. Sci..

[52] Bertolone L, Shin HKH, Baek JH, Gao Y, Spitalnik SL, Buehler PW (2022). ZOOMICS: Comparative metabolomics of red blood cells from guinea pigs, humans, and non-human primates during refrigerated storage for up to 42 days. Front. Physiol..

[53] Donald AB, Cuddington Clayton T, Martindale Evan L, Pires Ivan S, Palmer Andre F (2020). Controlled polymerization and ultrafiltration increase the consistency of polymerized hemoglobin for use as an oxygen carrier. Bioconjugate Chem..

