# Supplementary material for: Safety and efficacy of human polymerized hemoglobin on guinea pig resuscitation from hemorrhagic shock
Source: Sci Rep. 2022 Nov 28;12:20480. doi: 10.1038/s41598-022-23926-y (PMC9703428; doi:10.1038/s41598-022-23926-y)
Supplement: Supplementary file 1 — Supplementary Information. [file 41598_2022_23926_MOESM1_ESM.pdf]

Safety and efficacy of human polymerized hemoglobin on guinea pig resuscitation from hemorrhagic shock

Cynthia R. Muller[1], Alexander T. Willians[1], Cynthia Walser [1], Allyn M. Eaker[1]; Jose Luis Sandoval[1]; Clayton T. Cuddington[2], Savannah Wolfe[2]; Andre F. Palmer[2], Pedro Cabrales[1*]

1-Department of Bioengineering, University of California, San Diego, CA, USA.

2-William G. Lowrie Department of Chemical and Biomolecular Engineering, The Ohio State University, Columbus, OH, USA.

Supplementary Table 1. ELISA kits used in the analysis.

Method	Kit/Assay	Analyte	Vendor
ELISA	KA1625	AST	Abnova Corp, Taiwan
ELISA	KA4189	ALT	Abnova Corp, Taiwan
ELISA	BMS625	IL-6	Thermo Fisher, Waltham, MA
ELISA	BMS629	IL-10	Thermo Fisher, Waltham, MA
ELISA	KB02-H2	Creatinine	Arbor Assays Inc, Ann Arbor, MI
ELISA	K024-H5	BUN	Arbor Assays Inc, Ann Arbor, MI
ELISA	ERCXCL1	CXCL1	Thermo Fisher, Waltham, MA
ELISA	MCA-155	Ferritin	Serotec, Oxford, UK
ELISA	BA-E-6600	Catecholamines	ImmunoSmol, France
ELISA	ERLCN2	Urine NGAL	Thermo Fisher, Waltham, MA
ELISA	ab235627	Bilirubin	Abcam, Cambridge, UK
Luminex technology /Miliplex Immunoassay	RECYTNMAG-65K	MCP-1	Millipore Corporation, Massachusetts, USA
Luminex technology /Miliplex Immunoassay	RECYTNMAG-65K	TNF-α	Millipore Corporation, Massachusetts, USA
ELISA	ab246529	Cardiac Troponin	Abcam, Cambridge, UK
ELISA	ab256398	CRP	Abcam, Cambridge, UK
ELISA	ab108797	ANP	Abcam, Cambridge, UK
